# Investigating the impact of artificial intelligence on human resource functions in the health sector of China: A mediated moderation model

**DOI:** 10.1016/j.heliyon.2023.e21818

**Published:** 2023-11-10

**Authors:** Muhammad Farrukh Shahzad, Shuo Xu, Waliha Naveed, Shahneela Nusrat, Imran Zahid

**Affiliations:** a College of Economics and Management, Beijing University of Technology, Beijing 100124, PR China; b Institute of Business & Management, University of Engineering and Technology, Lahore 54000, Pakistan; c College of Environment and Life Science, Beijing University of Technology, Beijing 100124, PR China; d Department of Mechanical Engineering and Technology, Government College University Faisalabad, Pakistan

**Keywords:** Artificial intelligence, Technological awareness, Personal innovativeness, Perceived risk, Social media influence, Human resource functions

## Abstract

Artificial intelligence (AI) is rapidly transforming the way human resources (HR) functions are carried out in the health sector of China. This study aims to scrutinize the impact of artificial intelligence on the human resource functions operating in the healthcare sector through technological awareness, social media influence, and personal innovativeness. Additionally, this study examines the moderating role of perceived risk between technological awareness and human resources functions. An online questionnaire was administered to human resources professionals in the health sector of China to gather data from 363 respondents. Partial least squares structural equation modeling (PLS-SEM), a statistical procedure, is implemented to investigate the hypothesis of the projected model of artificial intelligence and human resource functions. The research findings reveal that artificial intelligence significantly influences human resource functions through technological awareness, social media influence, and personal innovativeness. Furthermore, perceived risk significantly moderates the relationship between technological awareness and human resource functions. The findings of this study have important implications for HR practitioners and policymakers in the health sectors of China, who can leverage artificial intelligence technologies to optimize and improve organizational performance. However, its adoption needs to be carefully planned and managed to reap the full benefits of this transformative technology.

## 1 Introduction

Artificial intelligence technologies have been the most illustrious factor across various industries around the globe due to their ability to automate routine tasks, improve decision-making and increase efficiency [1]. In today's digital world, artificial intelligence (AI) has been involved in diverse operations of the functional departments of healthcare institutions in China, which led to a revolution in business. Such practices are becoming increasingly significant in the healthcare sector of China by providing new innovative opportunities for efficiency, automation, and innovation to the human resource (HR) functions, including in the recruitment & selection process, training & development, and performance management [2]. Even recruiting a talented pool of candidates and managing multifariousness has become a terrific challenge for healthcare institutions in China.

Consequently, implementing IA helps the healthcare sector automate miscellaneous aspects of talent acquisition, from screening resumes to onboarding qualified candidates to cope with such challenges [3]. A prior study [4] reported that artificial intelligence had become one of the top trends in the business world that influences the HR functions of diverse organizations in the expeditious talent acquisition process. Further, AI-powered virtual training assistants can provide personalized training to existing and new entrants based on their needs and learning styles. Such practices can help improve training programs' effectiveness and increase employee engagement [5].

A viable solution to improve HR operations in the Chinese hospital setting is artificial intelligence. The delivery of healthcare services and employee satisfaction can be improved by utilizing AI technologies in HR departments. However, conventional HR procedures may be time-consuming, resource-intensive, and occasionally ineffective in dealing with the healthcare industry's particular problems [6].

Information technology can help analyze employee performance data and identify patterns and trends. AI technology can assist in identifying potential performance issues and providing training and coaching to improve employee performance [7]. In a multifaceted and challenging hospital environment, technological awareness in healthcare institutions has significantly influenced HR management towards adopting sophisticated artificial intelligence that undertakes work activities previously carried out by humans [8]. China's healthcare sector is huge and developing quickly due to the country's large population and rising demand for high-quality medical care. The HR departments' responsibilities are recruiting, managing, and retaining healthcare experts and employees, which are essential to this industry [9].

However, technological awareness helps HR professionals to recruit and onboard competent candidates from different professional recruiting platforms such as Indeed and LinkedIn [10]. Quite a few studies signify that technological awareness opens the window of opportunity for HR managers to monitor the performance of the staff systematically and add their remarks regarding employees' performance electronically [11]. Furthermore, social media influences the top management of healthcare organizations to transform HR practices to improve the employee experience. In such a manner, social media can be a powerful tool for healthcare professionals looking to accept artificial intelligence to improve HR functions for long-term sustainability [12]. Because it made it convenient for employees to access valuable information and insights, create awareness and technical support, and, more specifically, connect HR professionals with employees and technical staff in the healthcare institutes [13].

A researcher [14] in the study reveals that personal innovativeness is a propensity to use new technological practices to improve HR functions. Recruit talented candidates who provide quality healthcare services to diverse patients and monitor their activities on digital platforms to reduce cost and time. Consequently, such practices motivate the management to develop new dynamic and innovative HR practices like innovative training & development programs that recover the overall performance of the HR functions in the healthcare industry [15]. However, with the proliferation of technological advancement in diverse industries, many research scholars have faced resistance from healthcare managers toward adopting such innovative practices due to the perceived risk associated with artificial intelligence and technological awareness [16].

In their study, a researcher [17] concluded the financial, social, performance, and privacy risks that negatively influence the behavior of top-level management of healthcare institutes toward implementing such technological practices to improve HR functions. Another study [18] highlighted that HR managers would be less likely to use artificial intelligence practices if they perceive the extensive risk involved, and their awareness, knowledge, and experience with particular technology may be restricted. However, health organizations worldwide have adopted technological tools and software to manage the functional departments professionally and systematically for long-term sustainability; therefore, few organizations are still in the adoption process [19].

The prior scholarships have identified the relationship between artificial intelligence, technological awareness, and its overall impact on HR functions in different sectors [4,[20], [21], [22]]. No one is used as a mediator, such as technological awareness, social media influence, and personal awareness, to implement artificial intelligence to improve the HR functions of the health sector. Therefore, a more extensive study is needed that comprehensively addresses the pros and cons of implementing IA in the healthcare industry and its impact on HR functions while mitigating the perceived risk through technological awareness. This novel study inspects the implementation of IA technology in the overall HR functions of healthcare institutes in China. Therefore, this study will help artificial intelligence developers, designers, and HR managers to understand artificial intelligence functions and develop strategies to manage errors and improve their functions. Therefore, the first objective of this study is to give the road to health sector organizations using IA technologies in their HR to improve progress. Second, this study uses technological awareness, social media influence, and personal awareness to attentive health sector employees as technological changes occur nowadays. Third, this study aims to implement IA and its effects on HR operations while optimizing perceived risk through technological competence.

Moreover, this empirical research study is well-structured, with a literature review comprising the study's theoretical background, hypothesis derivation, and fundamental conceptual framework of artificial intelligence and HR functions. Eventually, it also demonstrates the research methodology that includes the study's research design and instruments, results, and data analysis. Further, this study also discusses the theoretical and practical implications of the study. However, this empirical study's conclusion, limitations, and future research direction have been portrayed at the end of this paper.

## 2 Literature review and supporting theories

### 2.1 Technology acceptance model

The technology acceptance model (TAM) by Ref. [23] is an insightful and vigorous framework considering the technical frames influencing healthcare managers' behavior, adoption, and understanding of a particular technology. However, adopting new technology in healthcare institutes illustrates a decision-making process that demonstrates the factors for acceptance and resistance of HR managers towards technology [10]. However, TAM proposes that healthcare HR managers' intention to utilize a particular technology comprises their perceived usefulness (PU) and perceived ease of use (PEOU). That indicated crucial indicators of whether they would adopt new technology to improve HR functions such as talent acquisition process, performance management, training & development.

However, perceived usefulness refers to the extent to which an individual believes using a particular technology will enhance performance. On the other hand, perceived ease of use refers to the extent to which an individual believes that the easier a technology is to use, the more likely people are to adopt it [23,24]. This can include factors like user interface design, training & support, and accessibility. However, the TAM framework has been extensively implemented the model in numerous explanatory studies [25,26] of technology implementation. In their study, a researcher [27] comprehensively probes the social media influence and cognitive as new factors to improve the specificity of the traditional TAM framework of investigating HR managers' intention towards the adaptability of a particular technology in healthcare institutes. Furthermore, diverse research scholars have demonstrated diverse TAM frameworks in their empirical research studies [12,28,29].

### 2.2 Artificial intelligence and HR functions

Artificial intelligence refers to the emergence of computer systems that can carry out operations that typically require human intelligence, such as recognition of speech, making decisions, and processing natural languages [18]. IA has the potential to reform many industries and improve our lives nowadays. However, there are concerns about artificial intelligence's moral and social implications, such as latent bias, judgment, job displacement, and loss of privacy [30]. In recent years, organizations have increasingly adopted AI-based solutions to enhance their HR functions. This trend is anticipated to continue because artificial intelligence has many advantages that can help businesses streamline their HR procedures and make better judgments [31].

The development of artificial intelligence demands rigorous consideration and regulation to guarantee that its advantages are maximized while its risks are minimized [4]. Organizations should ensure that their artificial intelligence solutions are based on various unbiased data sets and routinely audit their systems for potential bias to reduce these risks. As AI-powered solutions progress, they might be able to carry out specific HR activities faster and more precisely than humans, which would result in job losses [19]. To address this issue, Administrations should concentrate on retraining and upgrading workers to take on new tasks and responsibilities that sound for a human touch. Artificial intelligence technologies can help organizations improve their HR processes, increase efficiency, and make more informed decisions [14].

Corporations can enhance their hiring, training and development, and performance management procedures thanks to the growth of IA technologies. In the past, hiring managers would have to spend hours sorting through applications and resumes to find qualified individuals [32]. However, with the advent of AI-powered recruitment tools, recruiters can automate many tasks, including resume screening, candidate sourcing, and even initial interviews. AI-powered recruitment tools can also use machine learning algorithms to analyze data from job postings and candidate profiles, allowing recruiters to identify the most relevant candidates quickly [33]. These techniques can also evaluate candidates' communication abilities and personality qualities using natural language processing (NLP) algorithms and help reduce recruitment bias by eliminating the need for human decision-making [34]. Past studies [35,36] highlighted that AI-powered learning tools supply healthcare professionals with the most recent knowledge and best practices to help them adjust their learning, enhance their performance, and improve the quality of care.

Artificial intelligence is also being used to improve performance management, which can analyze data from various sources, including electronic health records, patient satisfaction surveys, and employee feedback [35]. Healthcare organizations offer insights into their staff members' performance, highlighting their strengths and growth opportunities. This evidence can then be used to develop targeted performance improvement plans for individual employees [36,37]. As technology continues to develop in the HR operations of the health industry, we can anticipate even more creative use of artificial intelligence. Based on the above discussion, we hypothesized.

Hypothesis 1 Artificial intelligence has a positive relationship with HR functions.

### 2.3 Mediating role of technological awareness

Artificial intelligence can automate tasks, analyze large amounts of data, and make predictions, leading to advancements in various fields. Making technology more useable and accessible is one-way artificial intelligence might raise awareness of technology [38]. Virtual assistants with AI capabilities can make it simpler and more effective for people to communicate with technology. As a result, one may become more accustomed to technology and comprehend its possibilities better [39]. AI can process vast volumes of data and find patterns that may be challenging for humans to notice using machine learning methods. This approach may result in new understandings and breakthroughs in engineering, finance, and healthcare [40]. Additionally, artificial intelligence can boost the productivity and security of several businesses, including manufacturing and transportation [41].

A past study highlighted the importance of the automobile sector as self-driving cars use artificial intelligence to navigate roads and avoid accidents. In contrast, manufacturing plants use artificial intelligence to optimize production processes and reduce waste [1]. Artificial intelligence can improve technological awareness by increasing accessibility to technology, boosting human comprehension of difficult topics, and raising the efficiency and safety of numerous businesses [17]. Artificial intelligence is the capacity of machines to mimic human intelligence and carry out operations like understanding, solving problems, and decision-making that traditionally demand human cognition. Technological awareness refers to the knowledge, skills, and understanding of technology and its applications [42].

Technological awareness has transformed the way healthcare is delivered. As a result, HR professionals must be aware of the latest technological advancements to recruit, train and retain the best employees [43]. HR professionals may find and attract competent applicants more effectively by using their technological awareness. It is possible to post job openings and reach a larger pool of people using social media platforms and online job boards. Furthermore, HR professionals can use applicant tracking systems to streamline recruitment and manage candidate applications more effectively [44]. Technology awareness can be leveraged to give healthcare workers opportunities for training and development. E-learning platforms, webinars, and virtual classrooms can deliver training content and ensure that employees are up-to-date with industry trends and best practices [8]. Technology can assist HR personnel in providing feedback and monitoring employee performance [18].

HR professionals aware of the latest technological advancements will be better equipped to meet the changing needs of the healthcare industry and attract and retain the best talent. However, the successful implementation and adoption of artificial intelligence in HR functions depend on the level of technological awareness of the individuals involved [45]. HR managers and employees lack the knowledge and skills to understand and use artificial intelligence, and the technology may not be effectively integrated into their workflows [43]. Therefore, technological awareness plays a mediating role in the relationship between artificial intelligence and HR functions in the healthcare sector of China. Hence, we hypothesized.

Hypothesis 2 Technological awareness significantly mediates the relationship between artificial intelligence and HR functions.

### 2.4 Mediating role of social media influence

Personalization artificial intelligence algorithms can analyze user data such as search history, location, and behavior to personalize the content users see on their social media feeds. Social media helps users see more relevant and interesting content and increase engagement towards digital platforms [46]. Social media sites use artificial intelligence (AI) technologies to find and delete offensive or damaging information, such as hate speech or cyberbullying. Social media makes users' environments safer and more encouraging [5]. AI-powered chatbots are widely used on social media platforms to offer support and customer service. These bots can respond to frequently asked inquiries, make product suggestions, and provide users with individualized help [47]. Artificial intelligence algorithms can analyze user data to create targeted advertising campaigns, which helps ensure that users see ads relevant to their interests and needs, leading to increased engagement and better return on investment for advertisers [48].

A more positive and engaging atmosphere may be created for users thanks to artificial intelligence, which can also enhance the user experience on social media sites [49]. Healthcare organizations now have a strong tool for talent recruitment thanks to social media. Using websites like LinkedIn and Twitter, recruiters may connect with hiring managers and post career possibilities for job seekers. These platforms have improved recruitment in terms of rapidity, effectiveness, and cost [50]. Social media allows Healthcare workers easier access to training and development. Employees can access online training courses and webinars from any location, making it simpler to take advantage of professional development opportunities [7].

Additionally, social media can be used to monitor employee performance and provide criticism [51]. A manager can monitor staff social media activity to see who interacts with clients or spreads knowledge about the business. This data can be used in employee recognition and incentive programmers to thank employees for their contributions or to pinpoint areas that can benefit from additional training or support [13]. Healthcare organizations must also be aware of the integrity and authenticity of the material on social media sites. Social media may be a valuable tool for HR operations in the healthcare industry, but it must be handled carefully and properly [52]. The implementation of IA in HR functions of the health sector in China has been increasing in the modern age.

Deep learning, managing natural language, and robotics are examples of IA technologies used in various HR practices, including hiring and selection, training, and performance management. Automating this procedure using artificial intelligence technologies will result in more accurate and effective evaluations of job candidates [53]. A past study highlighted [54] that social media can be used to promote employee engagement and training. Health sector employers in China can use social media platforms to share educational materials and to foster communication and collaboration among employees. Employers have leveraged social media to enhance their use of AI in HR functions, improving their ability to attract and retain top talent and enhancing the overall performance of their workforce [20]. Therefore, social media influence plays a significant role in mediating the relationship between IA and HR functions in the health sector of China. Hence, we hypothesized.

Hypothesis 3 Social media influence significantly mediates the relationship between artificial intelligence and HR functions.

### 2.5 Mediating role of personal innovativeness

Personal innovativeness states an individual's willingness and capability to adopt and use new technologies or innovations. In the context of artificial intelligence in healthcare, personal innovativeness can influence how healthcare providers and organizations perceive and use technologies to improve patient outcomes and optimize healthcare processes [20]. Increased willingness to learn and experiment with new technologies with personal innovators are more likely to seek out and explore new technologies, including artificial intelligence, and to experiment with their potential applications in healthcare. Consequently, it can lead to the significant adoption of artificial intelligence technologies [55].

Personal innovators intend to adopt change flexibly and are less likely to resist new technologies. Personal innovativeness can help to overcome potential barriers to artificial intelligence adoption, such as skepticism or resistance among healthcare providers [2]. Through innovativeness, individuals facilitate integrating IA knowledge into healthcare workflows and improve overall quality to improve healthcare processes and decision-making. Personal innovators could benefit from developing patient results, reducing healthcare costs, and improving complete care and treatment excellence [56]. Furthermore, a prior study [57] expressed personal innovativeness played a crucial role in driving the adoption of artificial intelligence in the healthcare sector and in realizing the potential benefits of these technologies for patients and healthcare organizations.

Personal innovation can assist HR managers in luring and retaining creative and innovative teams that produce fresh concepts to enhance healthcare procedures. HR professionals can leverage personal innovation to find staff members who are willing to learn and experiment with new things [58]. These workers can then receive training and development to improve their imaginative and creative problem-solving skills. Personal innovativeness can be used as an indicator to assess an employee's capacity for developing fresh concepts and solutions [59]. HR managers can use this information to reward and recognize innovative employees and to motivate others to be more innovative. Personal ingenuity can assist HR managers in identifying and developing a staff better suited to address the difficulties of the healthcare sector and enhance healthcare results [60]. An individual's capacity and readiness to absorb and employ novel concepts and technology are measured by their level of personal innovation.

Artificial intelligence has become a significant factor for HR functions in the health sector of China by automating routine tasks, such as data entry and analysis, and providing valuable insights into workforce management [61]. However, adopting artificial intelligence in HR practices requires a certain level of personal innovativeness from HR professionals. Innovative HR professionals with technical skills and knowledge are more willing to adopt artificial intelligence into HR functions effectively [62]. Highly innovative HR professionals are more likely to embrace artificial intelligence and effectively integrate it into their work, ultimately leading to improved HR functions and better healthcare outcomes. Hence, we hypothesized.

Hypothesis 4 Personal innovativeness mediates the relationship between artificial intelligence and HR functions.

### 2.6 Moderating role of perceived risk

Technological awareness refers to an individual's knowledge, understanding, and familiarity with technology. Similarly, perceived risk states an individual's awareness of the possible positive consequences of using a particular technology [63]. Concerns about privacy and security may lead individuals to hesitate to use certain online services or share personal information online, limiting their technological awareness [4]. Conversely, when individuals perceive a high risk-taking ability with technology, they may be more likely to be involved and develop a greater awareness and familiarity with it [64]. The perceived risk of using smartphones and social media has led to widespread adoption and high levels of technological awareness in those areas [65].

Perceived risk can be an important factor in shaping individuals' attitudes and behaviors toward technology and influencing their level of technological awareness. HR functions involve managing employees and their relationships with the organization, such as the talent acquisition process, training and development, performance management, and employee relations [21]. Individuals have a high level of technological consciousness and perceive the risks associated with using technology to be low. They may be more likely to adopt and fully utilize technology in their HR functions [19]. Risk-taking abilities of individuals could improve the efficiency and effectiveness of HR practices, leading to better outcomes for the health sector.

Perceived risk can effect an organization's ability to attract top talent. Potential candidates take risks using technology and uplift their industry [10]. Perceived risk can also impact the effectiveness of training and development programs [66]. Employees with a high perceived risk of speaking up about concerns or giving feedback may be less likely to do so. Perceived risk is also a personality element that can increase transparency and address performance issues [67]. Perceived risk impacts the organizations must prioritize creating a culture of trust, transparency, and clear communication with employees [68].

Technological awareness refers to the knowledge, understanding, and familiarity individuals or organizations have with new technologies. In the context of the health sector, this could include knowledge of electronic health records (EHRs), telemedicine, or other digital health tools [25]. In the healthcare sector, this could include practices related to recruitment, training, performance management, and employee engagement [69]. Perceived risk is the subjective evaluation of the potential positive consequences of adopting new technologies. This process includes concerns about data privacy, security, or the potential for errors or failures in the technology itself [70]. Furthermore, the authors [37] highlighted the complex interplay between technology, risk, and HR functions in the healthcare sector. Healthcare organizations must carefully weigh the potential risks and rewards of successfully adopting new technology in the health sector. They also need to build creative methods [71]. This study provides a procedure to mitigate perceived risks while maximizing the potential benefits of new technologies. Hence, we hypothesized.

Hypothesis 5 Perceived risk significantly moderates the relationship between technological awareness and HR functions.

### 2.7 Research questions and conceptual framework

**Fig. 1:**
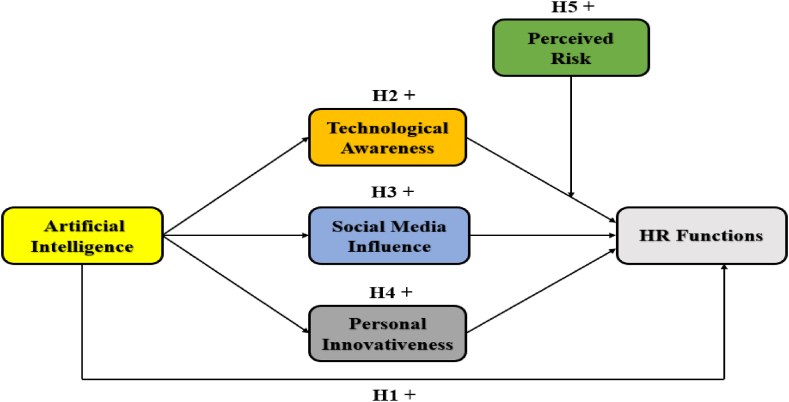
Conceptual framework.

The current study explains three research questions: first, does artificial intelligence link with HR functions of the health sector of China? Second, do technological awareness, social media influence, and personal awareness mediate the relationship between artificial intelligence link with HR functions? Third, does perceived risk moderate the relationship between technological awareness and HR functions? Furthermore, Fig. 1 below presents a conceptual framework after an in-depth literature review, as already mentioned above, to explain the model exhibited below.

## 3 Research methodology

This section extensively explains the research methods, survey instruments, data collection and sampling, demographic details, and procedure for conducting this empirical study. Foremost, we employed a quantitative research technique via an online survey method to gather the data for empirical analysis for this research study [72]. In today's digital world, gathering data from online channels is much faster, more efficient, and cheaper than the traditional dissemination of the manual questionnaire. Therefore, the best course of action is to acquire information through an online poll to get the respondents' responses. However, a convenience sampling technique is implemented for this cross-sectional preliminary research study to investigate the impact of artificial intelligence through technological awareness, social media influence, personal innovativeness, and perceived risk on the HR functions of healthcare organizations in China.

The quantitative method involves in this study discussing robustness, efficiency, ability to handle specific data types, and how it aligns with the research objectives. The fact that this strategy is suited for the study's sample size and data quality supports the claim that it is preferable. A deeper examination can be produced using SEM, a comprehensive statistical technique that enables you to evaluate and validate complicated correlations in your conceptual model. Choosing the most appropriate quantitative method is essential based on our research objectives, our conceptual model's complexity, and the target population's characteristics. Additionally, consider pre-testing your questionnaire to ensure clarity and reliability before deploying it on a larger scale. In quantitative research, researchers [21,22,73] demonstrate that firstly, research scholars must design the research instruments before data collection.

### 3.1 Data collection and sampling

Convenience sampling is a non-probability sampling method where researchers select the most readily available individuals or subjects. This study used convenience sampling to generate hypotheses or get initial insights. Secondly, this method allows researchers to collect data efficiently, which can be beneficial when time constraints are a significant consideration. Data is collected from human resource managers, operational managers, quality assurance managers, and other healthcare staff of government and non-government hospitals in China. Before gathering survey data, a cover letter was given to the hospital directors outlining the purpose, reassuring the confidentiality of respondents’ information that the data would only be used for research purposes, and seeking permission for data collection from the respondents.

### 3.2 Survey instrument development

Initially, we developed a questionnaire for the purpose of data gathering. However, a 5-point Likert scale is used to grade 31 items of respective constructs in the questionnaire, ranging from 1 (strongly disagree) to 5 (strongly agree). However, the questionnaire consists of two major sections. The first section includes demographic details, including age, gender, designation, and occupational experience. Respectively, the subsequent division is embedded with questions regarding artificial intelligence, social media influence, technological awareness, personal awareness, perceived risk, and human resource functions. This explanatory study includes six artificial intelligence items, as recommended by Ref. [74], to investigate their impact on the HR functions of healthcare institutes. Likewise, the study [47] suggested five items of social media influence to investigate the participants' response towards implementing particular technology through social media platforms to improve HR functions. However, the measurement scale of technological awareness is adopted from Ref. [10] with five items to demonstrate its influence on HR managers towards technology implementation. Further, five items of personal innovativeness are measured on a five-point Likert scale as endorsed by Ref. [75] to examine healthcare professionals' responses. Furthermore, five perceived risk items are being measured on a five-point Likert scale to investigate, manage and mitigate the degree of risk associated with implementing a particulate technology [76]. Finally, five items of human resources function are utilized from a research study conducted by Ref. [8] on a five-point Likert scale. All details of items are attached in Appendix A.

### 3.3 Respondent's summary

A convenience sample is considered to investigate the projected model of artificial intelligence and HR functions. However, the respondents of the present research study are HR managers, operation managers, quality assurance managers, and other healthcare staff of all functional departments of each government and a non-government medical institute in Beijing, China. The questionnaire survey is conducted, 400 questionnaires are sent out to the participants online, and 363 questionnaires are obtained with complete information from respondents. 37 responses were received with missing values. So, finally, we used n = 363 for analysis purposes. The response rate of the questionnaire survey is 90 %.

However, in this empirical analysis, we employed descriptive statistics. Although the survey data comprises 211 males (58 %) and 152 females (42 %), respondents demonstrate the ratio of participants between males and females who are influenced by artificial intelligence towards implementing a particular technology in HR. Therefore, a total sample of 363 healthcare service providers aged 20c44 is selected to participate in the present study for an empirical investigation. Although the ratio of respondents in terms of work experience with 1–5 years of experience is 38 %, with 6–10 years of experience is 44 %, and with 11–15 years of experience is 18 %. Likewise, HR managers 30 %, operation managers 30 %, quality assurance managers 33 %, and other healthcare staff 7 % are the ratio of respondents from whom we collected the data for conducting empirical analysis. Moreover, see Table 1, which reasonably outlines the respondent's demographic representation.

**Table 1:** Respondent's demographic characteristics.

Demographics	n = 363	Percentage (%)
Gender		
Male	211	58 %
Female	152	42 %
**Age**		
20–28 years	119	33 %
29–36 years	178	49 %
37–44 years	66	18 %
**Occupational Position**		
HR Manager	110	30 %
Operational Manager	108	30 %
Quality Assurance Manager	118	33 %
Other Healthcare Staff	27	7 %
**Professional Experience**		
≤1−5years	138	38 %
≤6−10yeras	160	44 %
≤11−15years	65	18 %
**Hospitals**		
Beijing Jishuitan Hospital	62	17 %
Beijing Henghe Hospital	57	16 %
Beijing United Family Hospital	68	19 %
Peking University People's Hospital	71	19 %
Beijing Hepingli Hospital	72	20 %
Beijing Goaxin Hospital	33	9 %

### 3.4 Common method and non-response bias

Research conclusions that rely on the survey data collection method may be influenced by common method bias [77]. Therefore, in this study, we employed several techniques to lessen the impact of common method bias. Since we only utilized one survey method to get the data, typical method bias can affect how reliable the study conclusions are. We employed anonymous respondents, well-designed questionnaires, and statements that accurately reflected both the questionnaires' positive and negative aspects, as the researcher recommended [77]. The total variation explained is less than 50 % according to data obtained using Harman's test using a single factor in factor analysis. This demonstrates that our study is free from common method bias.

Non-response is a challenge in survey research and impacts the findings. In this study, we examine early responders and late respondents divided at a ratio of 70:30 [78] on categorical variables (academic majors, place, and gender) in order to assess non-response bias. We use the *t*-test to compare the values of the variables in the early stage of the research and the late stage of the research to determine if non-response impacts the research outcomes. There was no difference between the two groups, according to the results (p-value >0.05). These test findings suggested that our research raised no questions about response bias.

## 4 Results

Partial least square structural equation modeling (PLS-SEM) is applied in the current study to evaluate the projected structure of AI and HR functions with Smart PLS 3.3 statistical software [79]. PLS-SEM has recently adopted a statistical method [80] to estimate the dysfunctional relationships among observed and latent variables [81]. PLS-SEM is principally suitable for the present study to operate [22,[81], [82], [83], [84]] diverse structure of mediation and moderation [85]. PLS-SEM amalgamates a two-step modeling procedure to determine the measurement and structure framework [86].

### 4.1 Measurement model evaluation

The measurement model of this study is validated by using the PLS-SEM approach. In the measurement model, confirmatory factor analysis CFA is executed on variables to validate the convergent validity [87]. The structure model validates such as internal consistency, discriminant validity, and goodness of fit in estimating [85]. Foremost, indicator reliability is inspected for validity. Likewise, Table 2 comprehensively exhibits the reliability values of each indicator that are greater than the minimum acceptable level of 0.4 or the estimated level of 0.7 [88]. Since prehistoric times, Cronbach's alpha has been used to evaluate internal consistent reliability [89]. Specifically, the items of the respective constructs are intended as valid items because the values of Cronbach's alpha are higher than the slightest satisfactory level of 0.7. Cronbach's alpha, values of artificial intelligence = 0.941, human resource functions = 0.873, perceived risk = 0.861, personal innovativeness = 0.953, social media influence = 0.935, technological awareness = 0.938. Even so, composite reliability is extensively applied for evaluation following the suggestion by Ref. [82]. All values of Cronbach's alpha are placed within the range in Table 2.

**Table 2:** Demonstration of FL, α , CR, AVE and VIF.

Latent constructs	Items	FL	α	CR	AVE	VIF
**Artificial Intelligence (AI)**			0.941	0.953	0.772	
	AI 1	0.851				2.687
	AI 2	0.887				3.333
	AI 3	0.900				2.443
	AI 4	0.852				1.912
	AI 5	0.894				3.145
	AI 6	0.885				2.756
**Personal Innovativeness (PINN)**			0.953	0.964	0.843	
	PINN 1	0.898				2.766
	PINN 2	0.941				3.295
	PINN 3	0.896				3.145
	PINN 4	0.946				1.422
	PINN 5	0.909				2.297
**HR Functions (HRF)**			0.873	0.907	0.665	
	HRF 1	0.709				2.511
	HRF 2	0.826				2.090
	HRF 3	0.881				2.317
	HRF 4	0.833				1.977
	HRF 5	0.817				2.581
**Technological Awareness (TA)**			0.938	0.953	0.803	
	TA 1	0.916				2.406
	TA 2	0.920				3.168
	TA 3	0.914				2.050
	TA 4	0.861				2.257
	TA 5	0.847				2.934
**Social Media Influence (SMI)**			0.935	0.951	0.793	
	SMI 1	0.887				2.511
	SMI 2	0.909				1.678
	SMI 3	0.897				2.563
	SMI 4	0.912				1.851
	SMI 5	0.847				2.651
**Perceived Risk (PR)**			0.861	0.898	0.641	
	PR 1	0.782				2.511
	PR 2	0.827				1.678
	PR 3	0.802				2.563
	PR 4	0.872				3.851
	PR 5	0.709				2.651

**Note(s):** FL = Factor loadings, VIF = Variance inflation factor, CR = Composite reliability, AVE = Average variance extracted, α = Cronbach's alpha.

The composite reliability in Table 2 comprehensively demonstrates the satisfactory values that disclose the extreme internal consistency reliability of ≥0.7 [73]. Composite reliability values of artificial intelligence = 0.953, human resource function = 0.907, perceived risk = 0.898, personal innovativeness = 0.964, social media influence = 0.951, technological awareness = 0.953. All values of Cronbach's alpha are positioned within the range in Table 2. Correspondingly, the average variance extracted prohibits the construct's convergent validity. Convergent validity is a common subject in the outer loading of each variable, and it is also measured from the average variance extracted (AVE) from each variable [90]. Nevertheless, from Table 2, the values of AVE are higher than the minimum acceptance value of 0.5 [91].

AVE values of artificial intelligence = 0.772, human resource function = 0.665, perceived risk = 0.641, personal innovativeness = 0.843, social media influence = 0.793, technological awareness = 0.803. All values of AVE are positioned within the range in Table 2. Consequently, the variance inflation factors VIF are employed for scrutinizing the multicollinearity [73]. A researcher [92] in a research study concludes that multicollinearity only appears when the values of VIF are higher than 5, which is high enough. Conversely, the consequences of the present research study demonstrate the value of VIF, as verified in Table 2, which is lower than 5 and forbids the scarcity of multicollinearity [88].

However, the PLS-SEM algorithm specifically measures the validity of the constructs. The discriminant validity ensures that every variable is distinct from the other variables [93]. Hence, the discriminant validity is evaluated by each latent variable's square root average variance extracted (AVE), which must be higher than the latent variable correlations. More specifically, Table 3 comprehensively exposes the present research findings that the model has satisfied the discriminant value. Second, the discriminant validity has been tested using the HTMT ratio and other cutting-edge methods. The information indicates that the HTMT ratio is below 0.85 [94]. Table 4 demonstrates very good discriminant validity.

**Table 3:** Discriminant validity through Fornell-Larcker criterion.

Construct	Mean	STDEV	1	2	3	4	5	6
**1. AI**	4.089	0.654	**0.879**					
**2. HRF**	4.097	0.589	0.461	**0.815**				
**3. PR**	4.339	0.639	0.363	0.339	**0.801**			
**4. PINN**	3.829	1.034	0.496	0.388	0.211	**0.918**		
**5. SMI**	4.292	0.817	0.299	0.408	0.399	0.119	**0.891**	
**6. TA**	4.073	0.635	0.497	0.494	0.281	0.445	0.333	**0.895**

**Note(s):** AI = Artificial intelligence, HRF = Human resource function, PR = Perceived risk, PINN = Personal innovativeness, SMI = Social media influence, TA = Technological awareness. Additionally, the square root of AVE is displayed on the diagonal and printed in bold.

**Table 4:** Discriminant validity through HTMT.

Construct	1	2	3	4	5	6
1. AI						
**2. HRF**	0.511					
**3. PR**	0.387	0.372				
**4. PINN**	0.522	0.424	0.223			
**5. SMI**	0.317	0.447	0.434	0.122		
**6. TA**	0.524	0.533	0.295	0.467	0.351	

**Note(s):** AI = Artificial intelligence, HRF = Human resource function, PR = Perceived risk, PINN = Personal innovativeness, SMI = Social media influence, TA = Technological awareness.

### 4.2 Structural model evaluation

**Fig. 2:**
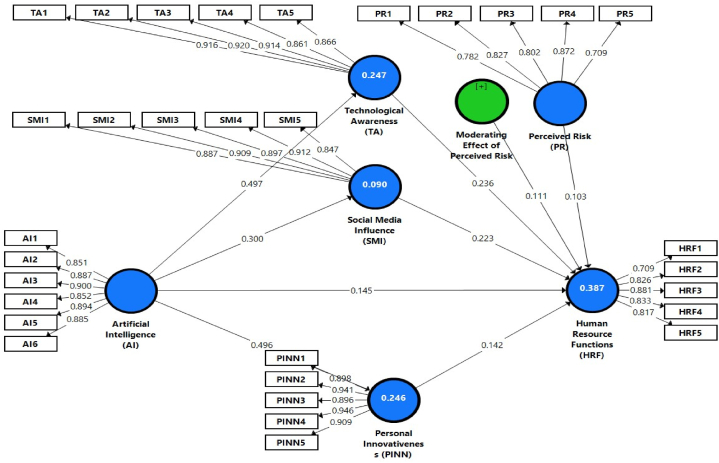
Valid estimated model (PLS Algorithm Diagram).

The structure model uses a bootstrapping technique with 5000 resamples used to obtain population mean (μ), standard deviation (STDEV), βeta -values, *t*-test values, and p-test values to authenticate statistical significance [95]. The R^2^ analysis estimates the coefficient of determination using the product of the dependent variable's variance ratio and the independent construct's total variance [96]. Values of R^2^ reflect how well the IVs are able to describe the DVs. Correspondingly, the values of R^2^ of human resource functions = 0.387, technological awareness = 0.247, social media awareness = 0.090, and personal innovativeness = 0.246. Furthermore, the difference between the examined interrelationship and the model indirect interrelationship matrix is known as the standardized root mean square residual (SRMR). Values less than 0.10 or 0.08 indicate a good fit in SRMR. The structural model is created using SmartPLS, and model fitness is examined. The model is usually well-fitting, according to the results of the fitness indices. As an illustration, the following values are given: SRMR = 0.0913, Chi-square = 6505.884, and NFI = 0.573. Fig. 2 represents the graphical representation of the structure model of artificial intelligence and HRF.

Furthermore, Table 5 points out the -values, p-test values, and path coefficients as generated by structure equation modeling (SEM) for hypothesis testing. Consequently, the present study further highlighted that artificial intelligence in the medical sector has an affirmative influence over improved HR functions with the values (β=0.145,t=2.601,p<0.05). So, this study's findings have supported H1. The study outcomes align with the literature on the relationship between artificial intelligence and Chinese health sector HR functions [2,97]. Similarly, artificial intelligence and technological awareness show a significant relationship with values (β=0.497,t=12.095,p<0.05). Technological awareness is positively related to HR functions with values (β=0.236,t=4.216,p<0.05). Further, artificial intelligence and social media influence have a significant relationship with the values (β=0.300,t=6.253,p<0.05). Social media influence is positively related to HR functions with values (β=0.223,t=4.266,p<0.05). Subsequently, a significant correlation exists between artificial intelligence and personal innovativeness with values (β=0.496,t=13.611,p<0.05). Personal innovativeness is positively related to HR functions with values (β=0.142,t=2.761,p<0.05).

**Table 5:** Hypothesis testing.

Paths	β-values	Mean	STDEV	t-values	p-values	Decision
Direct Effects						
AI → HRF	0.145	0.141	0.055	2.601	0.009	Supported
AI → PINN	0.496	0.496	0.036	13.611	0.000	Supported
AI → SMI	0.300	0.301	0.047	6.253	0.000	Supported
AI → TA	0.497	0.497	0.041	12.095	0.000	Supported
PR → HRF	0.103	0.109	0.051	2.012	0.040	Supported
PINN → HRF	0.142	0.141	0.051	2.761	0.006	Supported
SMI → HRF	0.223	0.225	0.052	4.266	0.000	Supported
TA → HRF	0.236	0.235	0.056	4.216	0.000	Supported
**Indirect Effects**						
AI → PINN → HRF	0.071	0.070	0.025	2.730	0.006	Supported
AI → SMI → HRF	0.067	0.067	0.018	3.632	0.000	Supported
A1 → TA → HRF	0.118	0.117	0.029	3.984	0.001	Supported
**Moderation Effects**						
PR*TA → HRF	0.111	0.120	0.051	2.147	0.032	Supported

**Note(s):** AI = Artificial intelligence, HRF = Human resource function, PR = Perceived risk, PINN = Personal innovativeness, SMI = Social media influence, TA = Technological awareness.

More specifically, the significant mediation role of technological awareness, social media influence, and personal innovativeness between artificial intelligence and HR functions in the healthcare industry is shown in Table 5. Firstly, technological awareness significantly mediates the relationship between artificial intelligence and 10.13039/501100014832HR functions with values (β=0.118,t=3.984,p<0.05). The findings of this study supported H2. The study outcomes align with the literature on the relationship between technological awareness mediates the relationship between artificial intelligence and HR functions [43,44]. Secondly, social media influence significantly mediates the relationship between artificial intelligence and 10.13039/501100014832HR functions with values (β=0.067,t=3.632,p<0.05). The results of this study supported H3. The study outcomes align with the literature on the relationship between social media influence mediates the relationship between artificial intelligence and HR functions [52,98]. Thirdly, personal innovativeness significantly mediates the relationship between artificial intelligence and 10.13039/501100014832HR functions with values (β=0.071,t=2.730,p<0.05). The findings of this study supported H4. The study outcomes align with the literature on the relationship between personal innovativeness mediates the relationship between artificial intelligence and HR functions [20,57].

Additionally, perceived risk has a pessimistic alliance with HR functions (β=0.103,t=2.012,p<0.05). The moderating role of perceived risk significantly moderates the relationship between technological awareness and HR functions with values (β=0.111,t=2.147,p<0.05). The study outcomes align with the literature on the relationship between perceived risk moderates the relationship between technological awareness and HR functions [4,37]. The results of this study supported H5. Thus, all hypotheses are accepted.

### 4.3 Moderating graph

Perceived risk as a moderating variable transforms the effectiveness and the path of the relationship of the independent and dependent variables in the structure model. Therefore, to test and scrutinize the impact of the moderation variable on the independent variable, mediating variable, and dependent variable by implementing the product indicator technique. However, Table 5 shows a moderation effect of perceived risk (β=0.111,t=2.147,p<0.05) to predict the appropriate level of technological awareness among HR professionals toward artificial intelligence implementations, which ultimately predicts the HR functions of healthcare managers.

**Fig. 3:**
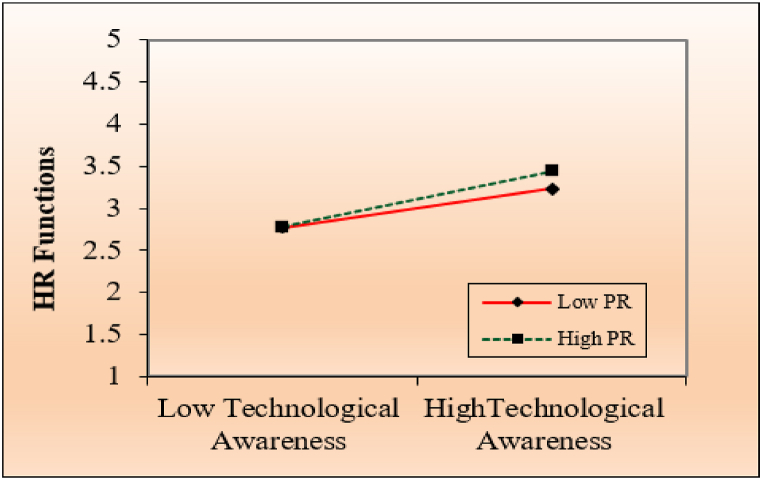
Moderating graph of PR between TA and HR functions.

The moderation effect of perceived risk is delineated in Fig. 3 at the two diverse respective levels. According to the slope, the higher the perceived risk, the higher the technological awareness, ultimately leading to improved and efficient HR functions. Conversely, the lower the slope of perceived risk, the lower the technological awareness among HR professionals. However, the higher perceived risk associated with higher technological awareness of the influence of artificial intelligence implementation on HR functions is strong. Therefore, low perceived risk leads to low technological awareness and weakens its impact on HR functions. Moreover, Table 5 comprehensively illustrates the moderation effect of perceived risk in forecasting technological awareness and HR functions. For a clear understanding, see Fig. 3, which represents the analytical representation of the moderation effect on the proposed model of artificial intelligence and HR functions.

## 5 Discussion and key findings

In the twenty-first century, the internet has evolved into a practical information-sharing platform, and the development of artificial intelligence has revolutionized traditional systems in various industries. AI has the probability of revolutionizing HR functions by systematizing repetitive tasks and providing data-driven perceptions with the help of AI health sector policymakers, enhancing abilities and leading to more effective HR strategies. The primary objective of conducting this research is to inspect the impact of artificial intelligence on HR duties like the hiring and selection process, training and development, and performance management in the health sector of China. This study also inspects the mediating role of technological awareness, social media influence, and personal innovativeness. Additionally, it inspects the moderating role of perceived risk between technological awareness and HR functions. Data was gathered from 363 employees who are working in hospitals in different positions. The questionnaires were given to the targeted sample for their opinion about artificial intelligence use in HR functions.

The results are deliberated in five major portions: first, the findings indicate that artificial intelligence substantially influences HR functions. Artificial intelligence is revolutionizing HR processes by advancing hiring and talent acquisition, boosting employee experience and improving employee engagement, enabling decision-making, facilitating employee development and upskilling, and automating HR operations [99]. A previous study [100] showed that adopting IA can streamline 10.13039/501100014832HR processes, optimize workforce management, and improve organizational performance, supporting our findings. Another past study [100] findings supported our study arguments as AI algorithms that analyze employee skill sets and career aspirations to recommend relevant learning opportunities to meet changing business demands.

Second, the current study investigated the mediating constructs of technological awareness between artificial intelligence and HR functions. The outcomes of this study show that the association between artificial intelligence and HR functions is significantly mediated by technological awareness, which is confirmed by the prior literature [41]. A past study [101] also highlighted that technological awareness referred to HR professionals understanding how artificial intelligence works, the data it uses, and how it can be integrated into existing HR processes. It can also help them identify potential ethical concerns and ensure that artificial intelligence is used fairly and unbiasedly. Through the help of technological awareness, HR professionals communicate more effectively with IT professionals and vendors responsible for implementing and maintaining artificial intelligence systems [40]. Similarly, a prior study supported our arguments as they evaluated and selected artificial intelligence solutions to align with their 10.13039/501100014832HR objectives, implement them, and train 10.13039/501100014832HR staff to utilize the technology effectively [102].

Third, the current research inspected the mediating role of social media influence between artificial intelligence and HR functions. The study findings illustrate that the affiliation between artificial intelligence and HR functions is significantly mediated by social media influence established by the prior literature [52]. A prior study highlighted that with the help of AI, HR departments analyzed social media data to identify latent job candidates and assess their fit with the organization [53]. Another study [103] backed our arguments as AI-powered sentiment analysis tools monitored employee sentiment on social media platforms, providing HR insights into employee morale and job satisfaction. A past study [53] focused on artificial intelligence to help HR professionals monitor social media channels, analyze feedback, and respond to real-time comments and reviews company's brand image. By combining the power of artificial intelligence with social media, HR can create a more efficient, effective, and engaging workplace.

Fourth, the present study investigated the mediating role of personal innovativeness between artificial intelligence and HR functions. The research outcomes express that the connection between artificial intelligence and HR operations is significantly mediated by personal innovativeness, confirmed by the prior literature [62]. Personal innovativeness refers to enthusiasm by an entity and the ability to implement novel concepts, technologies, and processes. Regarding HR operations, personal innovativeness can influence the degree to which personalities are willing to accept and use AI in the workplace [104]. Individuals who are more innovative and open to new ideas will likely embrace artificial intelligence in HR operations because they get potential benefits. Therefore, organizations looking to implement AI technology in their 10.13039/501100014832HR operations should consider their employees' level of personal innovativeness and provide training and support to help individuals become more innovative and adaptable [105].

Fifth, this study considered the moderating role of perceived risk between technological awareness and HR functions. The results show that the relationship between technological awareness and HR functions is significantly moderated by perceived risk, which is established by the prior literature [4]. A past study [70] highlighted how perceived risk influenced HR professionals using technology for recruitment. They are more engaged in adopting new technological solutions with high technological awareness. Another study [37] focused on perceived risks associated with technology adoption by controlling negative impacts such as data breaches, loss of human touch in employee interactions, and potential job losses due to automation. HR professionals with high awareness and risk-taking abilities are ready to adopt artificial intelligence [48]. This study's results show that strong evidence supports a higher perceived risk linked to greater technological awareness of the impact of artificial intelligence implementation on 10.13039/501100014832HR operations, from results and graphical representation in Table 5 and Fig. 3 that clearly depict that high risk associated with higher technology awareness which ultimately improves the execution of actions.

### 5.1 Theoretical implications

Artificial Intelligence has turned out to be a buzzword in today's digital world. However, the adoption of artificial intelligence has become widespread in various sectors. One major sector that has adopted artificial intelligence is the healthcare industry. Artificial intelligence has been applied to various HR practices, including talent acquisition process, training and development, and performance management, which have positively influenced the HR functions in the healthcare sector. This study has some theoretical implications. The study provides insights into the key factors that can facilitate or hinder the adoption and implementation of HR technology in the health sector of China.

Firstly, this research adds value to the existing pool of literature in numerous manners. This empirical study provides research indications of the positive influence of IA on HR functions in the healthcare sector of China. Second, the study highlights the role of technological awareness, social media influence, and personal innovativeness in facilitating the adoption of IA in HR functions. Third, specifically, the study highlights the moderating role of perceived risk between technological awareness and HR functions. This finding suggests that organizations should pay attention to the perceived risks associated with HR technology adoption and implementation to increase the chances of successful adoption and implementation. The findings of this study are reliable with the TAM model that posits that perceived usefulness and ease of use influence the intent to utilize innovation that ultimately influences behaviors towards the actual use of technology. The study extends the TAM by incorporating perceived risk as a moderator between technological awareness and HR functions.

### 5.2 Practical implications

This study also has some practical implications. The current research has several practical implications for HR managers in the healthcare sector of China. First, the study suggests that HR managers should know how artificial intelligence might improve HR processes, including performance management, training and development, and recruiting employees. Second, HR professionals should use social media to involve staff in adopting artificial intelligence and raise awareness of its advantages. Third, HR managers should encourage personal innovativeness among employees by providing training and development opportunities. Fourth, HR managers could address the perceived dangers of implementing IA by clearly outlining the advantages and hazards, ensuring data security, and fostering an environment of openness and trust. The study also highlights how technological awareness, social media influence, and personal innovativeness contribute to the literature on HR technology adoption and implementation. This finding suggests that to maximize the likelihood of effective adoption and execution, organizations should pay attention to the perceived risks associated with HR technology adoption and implementation. HR managers in the healthcare sector of China should be aware of these factors and take appropriate measures to leverage the benefits of artificial intelligence in HR functions.

## 6 Conclusion

### 6.1 Policy implications

In recent years, the health sector of China has shown a rising interest in adopting artificial intelligence in various aspects of their operations, including HR procedures, just as the hiring and selection process, training and development, and performance management. This empirical research intended to investigate the influence of IA implementation on HR responsibilities like talent acquisition process, training and development, and performance management in the health sector of China through technological awareness, social media influence, and personal innovativeness. Additionally, it inspects the moderating role of perceived risk between technological awareness and HR functions.

The research study has a five-fold contribution; at first, AI significantly influences HR functions in the health sector of China. Second, technological awareness significantly mediates the association between IA and HR functions. Third, social media influence significantly mediates the connection between IA and HR functions. Fourth, personal innovativeness significantly mediates the relationship between IA and HR functions. Fifth, perceived risk significantly moderates the relationship between technological awareness and HR functions. The research findings of this empirical research study indicate that artificial intelligence has a substantial potential to expand the proficiency and usefulness of HR functions in the health sector of China. However, organizations need to manage perceived risks associated with artificial intelligence adoption to maximize the benefits in their organizations. Moreover, social media influence and personal innovativeness are critical in determining the level of artificial intelligence adoption in HR functions. Healthcare organizations must foster collaboration between HR professionals and technology experts to ensure the effective adoption and utilization of artificial intelligence in HR functions.

### 6.2 Limitations and future directions

The current study comprises numerous research limitations that should be addressed in future research. Foremost, the present research focuses on the health sector of China, limiting its generalizability to other countries and sectors. Future research should also compare the findings across different countries to identify cultural and contextual factors that may impact the relationship between technological awareness and HR functions. Secondly, the limited sample size of this study may not represent the entire population of the health sector in China. Future research should use longitudinal designs or mixed methods to track changes over time and establish causality between technological awareness and HR functions. Thirdly, this cross-sectional empirical study limits the capability to inaugurate causality and track changes over time. Fourthly, the study does not control the other factors that may impact the relationship between technological awareness and HR functions, organizational culture, leadership, and employee attitudes. Future research should look for such competitive constructs that strongly impact technological awareness and HR functions, including organizational culture, leadership, and employee attitudes. Fifth, in this study, we collected data only from Beijing hospitals due to a shortage of time. For future researchers, they will consider different cities or draw comparisons. Lastly, in this study, risk acts as a moderator as people are more aware they are taking high risks and using new technology. For future perspective, researchers will use training and development of employees as important factors with awareness to use artificial intelligence.

## Funding

This work received financial support from the 10.13039/501100001809National Natural Science Foundation of China under grant number 72074014.

## Data availability statement

Data will be made available on request.

## CRediT authorship contribution statement

**Muhammad Farrukh Shahzad:** Writing – review & editing, Writing – original draft, Visualization, Validation, Software, Resources, Project administration, Methodology, Investigation, Formal analysis, Data curation, Conceptualization. **Shuo Xu:** Writing – review & editing, Writing – original draft, Supervision, Resources, Investigation, Funding acquisition, Conceptualization. **Waliha Naveed:** Resources, Methodology. **Shahneela Nusrat:** Writing – original draft, Project administration. **Imran Zahid:** Visualization, Supervision.

## Declaration of competing interest

Shuo Xu reports financial support was provided by 10.13039/501100001809National Natural Science Foundation of China. If there are other authors, they declare that they have no known competing financial interests or personal relationships that could have appeared to influence the work reported in this paper.

## Figures and Tables

**Table:** 

Constructs	Items		Sources
Artificial Intelligence (AI)	AI1	I have a sound knowledge of what artificial intelligence is.	[74]
	AI2	The implementation of IA in hospitals is capable of improving clinical decision-making.	
	AI3	Applying IA in hospitals could progress the delivery of direct easygoing care.	
	AI4	In the near future, IA may take over my healthcare professional designation.	
	AI5	An ethical principle is in place for the application of IA in the healthcare sector.	
	AI6	IA will reduce healthcare waiting times.	
Social Media Influence (SMI)	SMI1	Social media platforms allow HR managers to stay connected with other healthcare staff.	[47]
	SMI2	Social networks allow healthcare professionals with similar interests to stay connected and get advanced technical knowledge.	
	SMI3	Social networking sites have great potential for HR professionals.	
	SMI4	The development of social networking sites illustrates a growing need to apply IA in the healthcare sector.	
	SMI5	HR managers may use the information on social networking pages to make viability decisions.	
Technological Awareness (TA)	TA1	I place great importance on being the first to purchase new technology.	[10]
	TA2	I enjoy making high-tech purchases before most other people are aware of them.	
	TA3	Being the first to procure a high-tech item gives me a rush.	
	TA4	I wish to be the owner of cutting-edge technology goods.	
	TA5	I frequently purchase new technology when I see it on the market because it is new.	
Personal Innovativeness (PI)	PINN1	I appreciate myself for grasping the chances.	[75]
	PINN2	I would love to try out new technologies in hospitals to make things more efficient.	
	PINN3	New products are generally gimmicks.	
	PINN4	If I learned about an innovative technology, I would try to find a way to test it out.	
	PINN5	I am frequently the first to attempt the latest technologies among healthcare professionals.	
Perceived Risk (PR)	PR1	When IA is used, my particular data may drop into the wrong hands.	[76]
	PR2	It feels reservation when computers examine scans without the interference of humans.	
	PR3	IA makes healthcare staff lazy.	
	PR4	I think the standby of doctors by IA will materialize in the far future.	
	PR5	IA can only be executed to plaid human judgment.	
Human Resources Functions (HRF)	HRF1	IA for technology awareness is more cost-effective than other technologies.	[8]
	HRF2	IA technology helps HR managers to select the right candidates.	
	HRF3	IA technology helps HR managers to conduct online training and development sessions for new and existing employees.	
	HRF4	IA technology provides user-friendly mediums to monitor employees' performance.	
	HRF5	Tracking employees' activity through artificial intelligence technology is more efficient and time-saving.	
