# Supplementary material for: Investigating the impact of artificial intelligence on human resource functions in the health sector of China: A mediated moderation model
Source: Heliyon. 2023 Nov 10;9(11):e21818. doi: 10.1016/j.heliyon.2023.e21818 (PMC10685199; doi:10.1016/j.heliyon.2023.e21818)
Supplement: Multimedia component 1 [file mmc1.docx]

**Investigating the impact of Artificial Intelligence on HR Functions in the Health Sector of China: A Mediated Moderation Model**

**Note:** I am conducting research as a student of PhD in Management Sciences and Engineering School of Economics and Management, Beijing University of Technology, Beijing China. I am conducting research on **“Investigating the impact of Artificial Intelligence on HR Functions in the Health Sector of China: A Mediated Moderation Model”.** You are requested to spare your precious time to complete this survey. Your specific answers will be completely anonymous & confidential, but your views, in combination with those of others, are extremely important. Your cooperation is highly appreciated. Thanks once again for your time and cooperation.

**Section-1 Personal Memoranda**

1. Name (Optional)------------------------------------------------------------------------
2. Phone No (Optional)-------------------------------------------------------------------
3. Email Address (Optional)---------------------------------------------------------------
4. Designation-------------------------------------------------------------------------------
5. Department--------------------------------------------------------------------------------
6. Experience of working--------------------------------------------------------------------
7. Qualification:

- Post-graduate-----------------------------------------------------------------------
- Professional Graduate--------------------------------------------------------------
- Graduate-----------------------------------------------------------------------------
- Any other----------------------------------------------------------------------------

1. Gender of Respondent:

- Male------------------------------------
- Female---------------------------------

**Section – 2 (Please reply all the research questions.)**

1. **Artificial Intelligence**

| **Encircle only one number from 1-5 that indicates your disagreement or agreement** | | **Strongly**  **disagree** | **Disagree** | **Neutral** | **Agree** | **Strongly agree** |
| --- | --- | --- | --- | --- | --- | --- |
| AI1 | I have a sound knowledge of what artificial intelligence is. |  |  |  |  |  |
| AI2 | The implementation of IA in hospitals is capable of improving clinical decision-making. |  |  |  |  |  |
| AI3 | Applying IA in hospitals could progress the delivery of direct easygoing care. |  |  |  |  |  |
| AI4 | In the near future, IA may take over my healthcare professional designation. |  |  |  |  |  |
| AI5 | An ethical principle is in place for the application of IA in the healthcare sector. |  |  |  |  |  |
| AI6 | IA will reduce healthcare waiting times. |  |  |  |  |  |

1. **Social Media Influence**

| **Encircle only one number from 1-5 that indicates your disagreement or agreement** | | **Strongly**  **disagree** | **Disagree** | **Neutral** | **Agree** | **Strongly agree** |
| --- | --- | --- | --- | --- | --- | --- |
| SMI1 | Social media platforms allow HR managers to stay connected with other healthcare staff. |  |  |  |  |  |
| SMI2 | Social networks allow healthcare professionals with similar interests to stay connected and get advanced technical knowledge. |  |  |  |  |  |
| SMI3 | Social networking sites have great potential for HR professionals. |  |  |  |  |  |
| SMI4 | The development of social networking sites illustrates a growing need to apply IA in the healthcare sector. |  |  |  |  |  |
| SMI5 | HR managers may use the information on social networking pages to make viability decisions. |  |  |  |  |  |

1. **Technological Awareness**

| **Encircle only one number from 1-5 that indicates your disagreement or agreement** | | **Strongly**  **disagree** | **Disagree** | **Neutral** | **Agree** | **Strongly agree** |
| --- | --- | --- | --- | --- | --- | --- |
| TA1 | I place great importance on being the first to purchase new technology. |  |  |  |  |  |
| TA2 | I enjoy making high-tech purchases before most other people are aware of them. |  |  |  |  |  |
| TA3 | Being the first to procure a high-tech item gives me a rush. |  |  |  |  |  |
| TA4 | I wish to be the owner of cutting-edge technology goods. |  |  |  |  |  |
| TA5 | I frequently purchase new technology when I see it on the market because it is new. |  |  |  |  |  |

1. **Personal Innovativeness**

| **Encircle only one number from 1-5 that indicates your disagreement or agreement** | | **Strongly**  **disagree** | **Disagree** | **Neutral** | **Agree** | **Strongly agree** |
| --- | --- | --- | --- | --- | --- | --- |
| PINN1 | I appreciate myself for grasping the chances. |  |  |  |  |  |
| PINN2 | I would love to try out new technologies in hospitals to make things more efficient. |  |  |  |  |  |
| PINN3 | New products are generally gimmicks. |  |  |  |  |  |
| PINN4 | If I learned about an innovative technology, I would try to find a way to test it out. |  |  |  |  |  |
| PINN5 | I am frequently the first to attempt the latest technologies among healthcare professionals. |  |  |  |  |  |

1. **Perceived Risk**

| **Encircle only one number from 1-5 that indicates your disagreement or agreement** | | **Strongly**  **disagree** | **Disagree** | **Neutral** | **Agree** | **Strongly agree** |
| --- | --- | --- | --- | --- | --- | --- |
| PR1 | When IA is used, my particular data may drop into the wrong hands. |  |  |  |  |  |
| PR2 | It feels reservation when computers examine scans without the interference of humans. |  |  |  |  |  |
| PR 3 | IA makes healthcare staff lazy. |  |  |  |  |  |
| PR4 | I think the standby of doctors by IA will materialize in the far future. |  |  |  |  |  |
| PR5 | IA can only be executed to plaid human judgment. |  |  |  |  |  |

1. **Human Resources Functions**

| **Encircle only one number from 1-5 that indicates your disagreement or agreement** | | **Strongly**  **disagree** | **Disagree** | **Neutral** | **Agree** | **Strongly agree** |
| --- | --- | --- | --- | --- | --- | --- |
| Please indicate the choice that accurately reflects your firm’s overall performance. | | | | | | |
| HRF1 | IA for technology awareness is more cost-effective than other technologies. |  |  |  |  |  |
| HRF2 | IA technology helps HR managers to select the right candidates. |  |  |  |  |  |
| HRF3 | IA technology helps HR managers to conduct online training and development sessions for new and existing employees. |  |  |  |  |  |
| HRF4 | IA technology provides user-friendly mediums to monitor employees’ performance. |  |  |  |  |  |
| HRF5 | Tracking employees’ activity through artificial intelligence technology is more efficient and time-saving. |  |  |  |  |  |
